# Progress in the Application of Bronchoscopic Cryotherapy in Pediatric Pulmonary Diseases

**DOI:** 10.3390/children11091130

**Published:** 2024-09-18

**Authors:** Xiaofen Tao, Shuxian Li, Hujun Wu, Fang Jin, Guoping Jin, Lei Wu

**Affiliations:** 1 Department of Pulmonology, Children’s Hospital, Zhejiang University School of Medicine, National Clinical Research Center for Child Health, Hangzhou 310052, China; icyshiny@zju.edu.cn (X.T.); doctor_shuxianli@zju.edu.cn (S.L.); 6515115@zju.edu.cn (H.W.); 21218423@zju.edu.cn (F.J.); 2 Department of Endoscopy Center, Children’s Hospital, Zhejiang University School of Medicine, National Clinical Research Center for Child Health, Hangzhou 310052, China; 6200003@zju.edu.cn

**Keywords:** cryotherapy, bronchoscopy, pediatric pulmonary diseases

## Abstract

Cryotherapy in interventional bronchoscopy is a new treatment modality which has recently been made available for the pediatric airway. Lack of experience and safety concerns have led to hesitant adaptation. The aim of this work was to elaborate on the application progress of cryotherapy in pediatric pulmonary diseases and also to assess indications, success rates, and complications of airway cryotherapy in children. In summary, cryotherapy via flexible bronchoscopy is a safe and feasible method. The application scope in pediatrics is similar to that in adults, and it is mainly used in airway obstructive diseases. However, it is primarily caused by benign conditions, and the interventional treatment mainly seeks to provide long-lasting symptomatic alleviation. Thus, prevention, treatment, and prognosis of long-term complications are issues that should be carefully considered in pediatric applications.

## 1. Introduction

The physical treatment technique called cryotherapy employs low temperatures to target and destroy localized sick tissue. Herpes, nevi, and other body surface diseases were initially treated with cryotherapy [1,2]. The use of cryotherapy is becoming more common due to the ongoing advancements in endoscopic methods and medical equipment. Since Neel et al. [3] introduced tracheoscopy-guided cryotherapy for treating endotracheal tumors and in situ cancer in the 1970s, cryotherapy has become prominent in endotherapy. With its practical operation, high level of safety, and curative effect properties, cryotherapy has seen increasing application and development in adult respiratory intervention [4]. The first report on pediatric diagnostic flexible bronchoscopy was published in 1978 [5]. Children’s interventional bronchoscopy is crucial in the treatment of pediatric respiratory disorders. The application of endoscopic balloon dilatation, tumor resection, laser therapy, and cryotherapy in pediatrics is becoming increasingly widespread [6].

Cryoprobes that were as tiny as 1.1 mm have been developed, paving the path for their application in pediatrics of all ages and creating new possibilities for interventional pediatric pulmonary endoscopy. The most often reported interventional method was the use of cryoprobes (33% for cryoextraction and 18% for cryodebulking). The reason behind this could be that the 1.1 mm cryoprobe (ERBE USA, Inc., Marietta, GA, USA) has made cryotherapy more widely used in pediatrics because it can now fit through bronchoscopes with 1.2 mm working channels, which are the most commonly used in pediatrics [7,8]. 

More research is required to assess the effectiveness and safety of cryotherapy in children because there are currently few literature reports on the subject and gaps in many areas. In order to create a comprehensive overview of the application and development of cryotherapy in the pediatric respiratory system, we conducted a study of the pertinent literature.

### 1.1. The Principle of Cryotherapy

Cryotherapy uses intense cold with rapid freeze–thaw cycles to destroy tissue. It was first used by Gage in 1968 on an endobronchial tumor. The technique uses a rigid applicator known as a cryoprobe [9]. This became increasingly popular with the application of the flexible fiberoptic cryoprobe [10]. Due to various techniques that are currently accessible, cryotherapy is applied in a number of clinical settings, including foreign body removal, treatment of low-grade airway malignancy, treatment of benign and malignant central airway obstruction, and transbronchial and endobronchial biopsies.

Extreme cold exposure causes cell death in tissue through several mechanisms: extracellular and intracellular ice crystals disrupt organelles and cause shifts of transcellular fluid (cell injury effect); local vasoconstriction and thrombosis result in ischemia (vascular injury effect); and possible immune-mediated cell death occurs (immunological effect) [11,12,13]. Ice crystal formation causes immediate and direct cell injury, while vascular and immunologic processes lead to delayed cell death [14]. For an area to be treated to achieve sufficient cell death (90%), it must be lowered to −40 °C at a rate of 100 °C per min [13,14,15]. The intracellular water content and vascularity of a tissue determine its cryosensitivity. High vascular tissues such as tumors, granulation tissue, skin, mucous membranes, nerves, and endothelium are cryosensitive, while fat, cartilage, nerve sheath, connective tissue, and fibrosis are cryoresistant [13]. 

The cryoprobe works on the Joule–Thomson effect, which states that as high-pressure gas expands through the hole, it absorbs a lot of heat from the environment and rapidly cools the probe head and tissue around it to produce the effects of cryotherapy. The three most often utilized coolants are carbon dioxide, nitrous oxide, and liquid nitrogen. The control device mainly includes the freezer host, control panel, and foot switch. Both rigid bronchoscopes and flexible bronchoscopes can be used to insert cryoprobes. 

Rigid bronchoscopes have large working channels, making it easier to handle situations such as massive hemoptysis, foreign bodies in large airways, and stent implantation, thereby ensuring airway safety. Although laser treatment can also be used under flexible bronchoscopes, the metal body structure of the rigid bronchoscopes is more helpful in reducing the risk of airway burning than the rubber outer skin of the flexible bronchoscopes. In addition, rigid bronchoscopes can be used to directly remove obstructive lesions in the cavity, shortening the operation time and improving efficiency. At the same time, mechanical ventilation can be performed simultaneously when using rigid bronchoscopes, which is more suitable for treating patients with severe tracheobronchial stenosis. Under rigid bronchoscopes, larger related accessories can be used to obtain more and larger biopsy tissues. Rigid bronchoscopes also have obvious advantages in dealing with operational complications, which can ensure airway patency and timely control of bleeding. The disadvantage of rigid bronchoscopes is that due to their large outer diameter, they cannot be bent, making it difficult to reach deeper bronchioles, and sometimes they are prone to incisor damage, laryngeal spasm, etc., so their application in distal airways is limited. Furthermore, most patients with rigid bronchoscopes require general anesthesia. In addition, rigid bronchoscopes are not suitable for patients with unstable necks, severe neck stiffness, or limited temporomandibular movements [16].

Compared to rigid bronchoscopes, flexible bronchoscopes have a smaller outer diameter and can be bent, allowing for deeper and finer areas to be observed intuitively and quickly for deep lesions. Due to its flexibility and softness, it has minimal damage for the diagnosis and treatment of distal airway lesions and can fully attract deep secretions for bronchoalveolar lavage. In addition, it can be operated under local anesthesia or general anesthesia with the use of a regular frequency ventilator to assist breathing. Therefore, the indications for flexible bronchoscopy are constantly expanding, and its clinical application is becoming increasingly widespread. The disadvantage of flexible bronchoscopy is that due to its small inner diameter, small endoscopic field-of-view, small clamping surface, and soft lumen, it is difficult to operate for large airway diseases [16]. 

Bronchoscopic cryotherapy includes frozen resection and freeze–thaw surgery. Frozen resection was proposed by Hetzel et al. [17] in 2004. It is performed with a sharp drop in local temperature after the probe contacts the target tissue. A portion of the lesion tissue is frozen on the freezing probe and torn from the airway in the frozen state. The probe and bronchoscope are quickly pulled out together with the lesion tissue, and the operation can be repeated. It is mainly used for cryobiopsy, and freezing and removing of tumors, foreign bodies, necrotic objects from the airway, etc. In the freeze–thaw method, the probe is placed on the lesion tissue. The lesion tissue is cooled to −60~−70 °C. Freezing treatment is continued for 1–3 min. Then, the lesion tissue is allowed to thaw naturally. The “freeze-thaw” cycle is repeated to achieve the treatment purpose. This is often used to treat traumatic airway stenosis, granuloma, tracheal/bronchial tuberculosis, etc. Airway scar stenosis can be first dilated with a balloon to expand the lumen and then combined with freezing and thawing to prevent restenosis. Freeze–thaw occurs when the local temperature falls after touching the probe, resulting in tissue cell necrosis and degeneration. While frozen cutting is quick, freezing and thawing are slow processes. Mucosal swelling and necrosis may occur after freezing, making them unsuitable for severe airway obstruction and urgent relief of airway obstruction [10,18]. Scar fibroblasts may be differentiated into normal fibroblasts by freezing, which will lessen the growth of scar tissue. This is a remarkable feature of cryotherapy after the repair of frozen damaged mucosa [19]. Thermal ablation therapy is, therefore, less effective than cryotherapy in the treatment of granulation tissue hyperplasia and scar contracture stenosis.

### 1.2. Bronchoscopic Cryotherapy Procedure

#### 1.2.1. Preoperative Preparation

##### Patient Preparation

A comprehensive assessment of the patient’s general condition and the risk of treatment is conducted. The chest X-rays and chest computed tomography (CT) scans are read regularly to familiarize oneself with the location, extent, length, and range of the lesion in order to determine whether to freeze–thaw or frozen-cut.

##### Preparation of Equipment

(1) Bronchoscopy: a. Rigid bronchoscope; b. Flexible bronchoscope.
(2) Cryotherapy device: Consists of three parts: cooling source, control device, and cryoprobe.

##### Freezing Equipment

**Figure 1:**
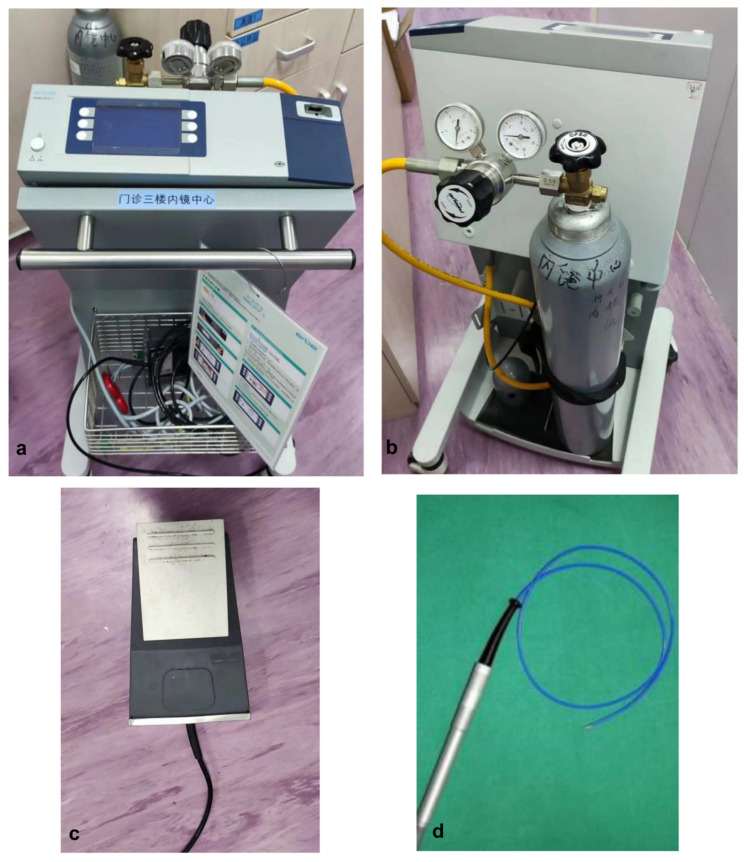
(**a**) Front view of the cryotherapy machine (ERBECRYO^®^ 2); the non-English term “门诊三楼内镜中心”: Third Floor Endoscopy Center. (**b**) Rear view of the cryotherapy machine (ERBECRYO^®^ 2); “内镜中心”: Endoscopy Center. Cryotherapy machine accessories: (**c**) foot pedal. (**d**) soft freezing probe.

(1) Three main types of coolants: carbon dioxide, nitrous oxide, and liquid nitrogen.
(2) Control device: mainly includes the freezing host, control panel, and foot switch.
(3) Freezing probes are divided into flexible freezing probes and rigid freezing probes (Figure 1).

#### 1.2.2. Operation Steps and Methods

(1) Patient preparation.
(2) Anesthesia: Local anesthesia or general anesthesia.
(3) The location and extent of the lesion are determined, and its surface secretions and accumulated blood are cleaned.
(4) The cryoprobe is inserted through the working channel, with the metal end at least 5 mm away from the distal end of the bronchoscope.
(5) The tip or sidewall of the probe can be used to freeze the lesion, and the metal tip should be placed as close to or deep into the lesion as possible. The cycle from freezing to thawing is 1–3 min, and each point should be repeatedly frozen and thawed 1–3 times. Several freezing points can be set for large lesions.
(6) About one week after the first stage of cryotherapy, bronchoscopy should be re-examined to evaluate the efficacy, the necrotic tissue should be cleared, and cryotherapy can be performed again for residual lesions.

Video S1 presents the procedure performed in our center.

#### 1.2.3. Matters Needing Attention

(1) Cryotherapy is mainly used to remove benign or malignant lesions in the airway cavity. Therefore, it cannot remove invisible tissues and is ineffective for extraluminal pressure lesions.
(2) When treating target tissues with freeze-thaw therapy, since the effect of one cryotherapy is not obvious, the same area should be subjected to at least three cycles of rapid freezing and slow thawing to achieve the maximum freezing effect. Tissue shedding may occur one week later, so it is not suitable for the treatment of lesions that are about to cause respiratory failure and require immediate removal. In addition, the possibility of asphyxia caused by the edema of surrounding tissues after freeze–thaw therapy should be considered. Freeze-cutting therapy is relatively fast, but for asphyxiating endotracheal lesions, it should still be carefully selected and not be the preferred technique.
(3) When using spray cryotherapy, consideration should be given to the rapid expansion of gas in a short period of time, so the effective channel for gas release should be unobstructed to avoid the occurrence of complications.
(4) Cryotherapy is only a treatment technique within the airway cavity, and its combination with other treatment methods can achieve more significant therapeutic effects.
(5) During the operation, the cryoprobe should be inserted through the working channel, and its metal end should be at least 5 mm away from the distal end of the bronchoscope to prevent damage to the bronchoscope.

## 2. Clinical Use of Bronchoscopic Cryotherapy

### 2.1. Application in the Diagnosis and Treatment of Benign Airway Obstructive Lesions

#### 2.1.1. Bronchial Foreign Body

**Figure 2:**
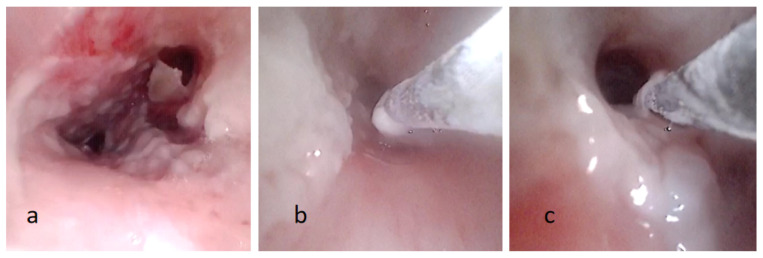
(**a**) Fiberoptic bronchoscopy image showing pseudomembranous necrotic substance on the tracheobronchial wall. (**b**,**c**) The process of removal pseudomembranous necrotic substance by cryotherapy.

A study on bronchoscopy in adults performed in 1997–2008 showed that 0.32% were due to aspiration of a foreign body [20]. Unlike adults, children frequently experience bronchial foreign bodies [21]. Besides regular foreign bodies, forceps can remove nuts and other irregular foreign bodies. However, cryotherapy is more effective at removing unique foreign bodies (such as pen caps and endogenous foreign bodies). It can drastically reduce the time needed to complete the procedure. Cryotherapy was employed by 8 out of 12 youngsters, according to Zhang et al., and the foreign body was effectively removed without any problems [22]. David et al. used a frozen probe to remove a pin successfully [23]. In a study by Han et al., between 2018 and 2021, 633 cases of foreign bodies were removed at their center, with 62 cases of foreign body aspiration being removed via combination cryotherapy, accounting for nearly 10% [24]. More interestingly, Li and Sun successfully removed a live leech from the airway using a cryoprobe [25]. In addition to exogenous foreign bodies, endogenous foreign bodies, such as plastic bronchitis, necrotic debris, blood clots, and so on, are also rather prevalent in pediatrics. Kallam et al. reported that cryotherapy was successfully utilized in two patients to remove the plastic sputum plug, which significantly reduced the symptoms of airway blockage [26]. Gatt et al. used cryotherapy to remove the blood clot successfully [27]. A study by Schramm et al. required additional instruments for foreign body retrieval [28]. The likelihood of success with cryoprobe is influenced by the characteristics of the foreign body and its water content, technical skills, location, and size of the airway [28,29]. Foreign bodies with low water content will have trouble adhering to the probe compared to those with higher water content. Recently, our center has successfully removed a pseudomembranous necrotic substance from a patient by using the frozen probe (Figure 2). The little frozen probe is more advantageous if the necrotic material is simple to break with biopsy forceps. Additionally, children between the ages of one and three have a high incidence of having a foreign body in their respiratory system. In children of this age, a relatively slim bronchoscopy needs to be used, with just a 1.2 mm working channel. The invention of the small frozen probe with an outer diameter of 1.1 mm will expand the applications for this device in removing foreign objects from the airways.

#### 2.1.2. Bronchial Tuberculosis

The type of tracheobronchial tuberculosis (TBTB) in children is primarily neoplastic (lymph node fistula). With localized airway mucosa rupture, granulation hyperplasia, and cascades, distal airway ventilation is often satisfactory [30]. Its therapeutic goals include effective caseous material removal, local granulation eradication, and airway smoothing. The main application of interventional treatment is cryotherapy, which can successfully remove caseous materials and granulation, and decrease the duration of bronchial TB therapy. Zhang et al. performed a retrospective study on eight cases of lymph node fistula, one case of inflammatory infiltration, and one case of granulation proliferative type TB, and found that transbronchial cryotherapy is safe and has good efficacy for TBTB in children, particularly for lymph node fistula or granuloproliferative type [31]. Zhao et al. reported seven cases of lymph node fistula TBTB; all were treated with CO_2_ cryotherapy combined with foreign body forceps and local injection drug treatment on the basis of systemic anti-tuberculosis chemotherapy, and found that CO_2_ cryotherapy can improve the treatment effect, reduce the occurrence of complications, and is safe and reliable for infants [32]. For bronchial tuberculosis with a protracted disease course and severe airway obstruction, holmium laser or argon plasma coagulation (APC) could also be chosen to clear the airway according to the condition under the bronchoscopy [33,34]. However, care should be taken to avoid damaging the normal tissue and structure of the airway when applied. Airway scar contracture, stenosis, or even occlusion can result from advancing bronchial TB in certain children. After balloon expansion, the patient may receive cryotherapy to prevent granulation or scar hyperplasia, or they may receive laser and high-frequency electro-knife therapy to treat the wound and base and remove granulation tissue and scar tissue that has protruded into the lumen. While treating tuberculous airway stenosis, consideration should be given to systemic antituberculosis therapy [33,34].

#### 2.1.3. Post-Traumatic Tracheobronchial Stenosis

**Figure 3:**
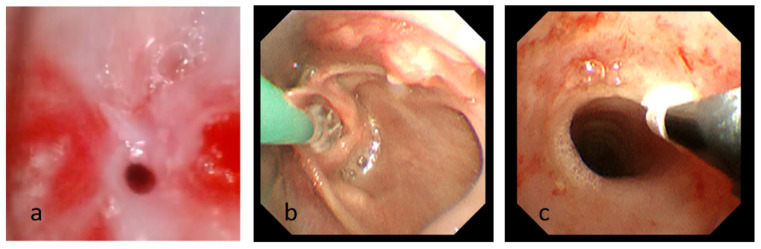
(**a**) Fiberoptic bronchoscopy image showing subglottic stenosis after intubation. (**b**) The process of balloon dilatation. (**c**) The process of cryotherapy Photograph showing the subglottic airway is significantly enlarged compared to before.

Granulation hyperplasia, scar contracture stenosis, with or without cartilage ring destruction caused by tracheal intubation, tracheostomy, and surgery are the major causes of post-traumatic tracheobronchial stenosis. Its pathogenic component may consist of one or more components. For tracheobronchial stenosis after trauma, such as subglottic stenosis after intubation, it is advised to use holmium laser ablation for granulation or scar tissue to obtain absolute lumen expansion, supplemented by repeated cryoablation to treat the wound surface and base because the local lumen in children is significantly smaller than that of adults. It is challenging to obtain promising long-term effects with balloon expansion alone [35]. Within 1 to 3 days after freezing and thawing, there is a high incidence of necrotic epithelium shedding. So, we need to pay close attention to the breathing condition of the children, recheck bronchoscopy in time, clear the necrotic epithelium, and unblock the airway. The freeze–thaw cycle depends on the repair status of the wound, and generally, there is no recurrence after a follow-up of 6 months, which can be considered a clinical cure. For treating benign pediatric airway lesions, Lee et al., Lawlor et al., and other researchers reported on the safety and efficacy of cryotherapy in pediatric airway granulation hyperplasia, respectively [36,37,38]. They also concurred that APC combined with CO_2_ cryotherapy could be used as one of the optional methods to remove granulation tissue and airway obstruction quickly. A study by Hosna et al. found that bronchoscopic cryotherapy can be an alternative treatment to laser surgery and tracheobronchial stenting for post-traumatic tracheal stenosis. Its procedure is relatively simple, low-risk, and cost-effective compared with other treatment approaches [39]. Our center also had experience in bronchoscopy intervention treatment of several cases of tracheal stenosis after intubation. It was found that balloon dilation combined with cryotherapy can obtain a better therapeutic effect (Figure 3).

#### 2.1.4. Benign Airway Tumors

The most common benign tumors of the airways in children are papillomas, hemangiomas, leiomyomas, and pleomorphic adenomas [40,41,42]. In recent years, cryotherapy has become particularly useful for treating laryngeal as well as tracheal and bronchial papillomas. For infantile hemangiomas, propranolol treatment is the first choice, while for hemangiomas with poor drug treatment effect, a high-frequency electric snare device or laser or high-frequency electric knife can be used for removal after blood supply vessel occlusion. The base can be treated with APC and freeze–thaw therapy [43,44,45]. One case of tracheal lobular capillary hemangioma was successfully managed by using an electric snare in our center [46], and there had been reports of cryotherapy for this disease in children [47,48]. In order to prevent the expansion of the extent of lesions brought on by thermal ablation treatment, the method of freezing and frozen cutting was utilized as much as feasible in treating laryngeal papilloma [49]. Forty-four benign endobronchial tumors with histological confirmation were summarized by Dalar et al., and all patients who had diode laser and argon plasma coagulation in combination with or without cryotherapy experienced tumor regression [50].

#### 2.1.5. Congenital Airway Stenosis

Wang reported a 23-month-old boy brought in for wheezing and hoarseness shortly after birth. The congenital laryngeal web was identified, and after carbon dioxide laser and cryotherapy under bronchoscopy, a favorable prognosis was given. Cryotherapy improved the mucosal smoothness [51].

#### 2.1.6. Granulation Hyperplasia Resulting from Other Causes

**Figure 4:**
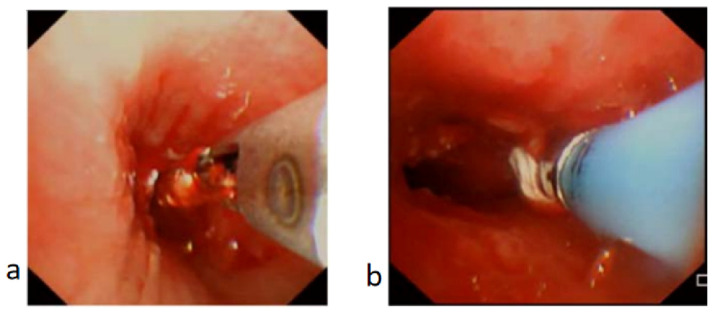
Granulation tissue in bronchus treated with biopsy forceps and cryotherapy. (**a**) Biopsy forceps applied to trim off the granulation tissue. (**b**) Cryotherapy applied to ablate the granulation tissue and minimize recurrence of granulation tissue or scarring.

Granulation hyperplasia can result from an infection, and airway stenting may lead to airway restenosis [52,53]. Granulation tissue hyperplasia after stenting can be treated with holmium laser, APC, CO_2_, cryotherapy, or balloon dilation. After stenting removal, the wound can be treated using CO_2_ cryotherapy to decrease the rate of granulation tissue hyperplasia [53,54]. Data from our center show that between January 2016 and December 2019, eight patients with granulation tissue hyperplasia brought on by bronchial foreign bodies underwent interventional treatment, seven of whom underwent carbon dioxide cryotherapy, with a 100% success rate and no complications [55] (Figure 4).

### 2.2. Interventional Diagnosis and Treatment of Malignant Tracheobronchial Tumors

Children seldom develop malignant tracheobronchial tumors, and among the pediatric airway malignant tumors described in the literature are leiomyosarcoma, inflammatory myofibroblastoma, and mucoepidermoid carcinoma [28,56,57,58,59]. Jieli et al. found that almost all children with tracheobronchial mucoepidermoid carcinoma were low-grade and intratracheal type, while most adults were invasive and high-grade [60].

The use of a bronchoscopic frozen small probe in diagnosing and treating malignant tracheobronchial tumors primarily entails tumor biopsy, removing the malignant tumor’s airway obstruction, palliative treatment of malignant tumors, and management of the airway prior to and following radical surgery [61,62]. To determine the risk of intraoperative bleeding, an enhanced CT scan should be carried out before the frozen biopsy of the tumor. Patients at risk of major bleeding need to undergo a biopsy after bronchial artery angiography and bronchial artery embolization. The best treatment for airway malignant tumors is surgery, but for tissues that cannot be operated on or remain after surgery, freeze–thaw therapy is used to reduce local recurrence.

### 2.3. Frozen Biopsy of Tracheobronchial or Intrapulmonary Lesions

In bronchoscopic cryobiopsy (CB), the tip of the cryoprobe is delivered to the pathological tissue in the bronchus or lung, and the heat of the surrounding environment is absorbed through the rapid release of coolant so that the cryoprobe can rapidly cool down and freeze the tissue around the probe. By using the frozen adhesive force, the probe and the frozen tissue around it are retracted as a whole to obtain the target tissue. It is divided into transbronchial lung cryobiopsy (TBLC) and endobronchial cryobiopsy (EBCB) [63,64].

German researcher Hetzel first validated the efficiency and safety of using the freezing approach for endobronchial lesion samples in 2008 [65]. In 2009, there was additional development of the freezing method for the biopsy of peripheral lung lesions by Babiak et al. [64]. Following that, the adult bronchoscopy frozen biopsy method quickly advanced. The frozen biopsy is barely mentioned in a few case studies and is still primarily exploratory in children [28,66,67,68]. Dhochak et al. initially described using the bronchoscopy cryobiopsy technique in children in 2021 [69]. They then described an empirical transbronchial, microscopic frozen biopsy in five children with diffuse lung illness [70]. Schramm et al. subsequently elaborated on the application prospects of cryobiopsy in pediatrics [28]. In bronchoscopic cryotherapy, a sufficient amount of tracheal histological specimens can be obtained for pathological examination through cryoablation, and hemostasis can also be performed through cryotherapy. Compared with the traditional tracheoscopy scrape biopsy and brush smear biopsy, cryobiopsy can obtain more histological specimens, reduce compression on specimens, and improve specimen quality without increasing the risk of complications such as airway bleeding [71]. The safety and efficacy of TBLC as a biopsy technique have been demonstrated in reasonably high-quality adult randomized studies [72,73], and the related guidelines of operation instructions are presented [74]. Cryobiopsy has been shown to be a safe and promising technique that can improve the histological diagnosis of childhood interstitial lung disease [75].

A thoracoscopy or thoracotomy lung biopsy is the gold standard for identifying interstitial lung disorders (ILDs). Although many patients with severe pulmonary interstitial disease cannot tolerate a surgical lung biopsy, it is challenging to make a conclusive diagnosis and determine the best course of treatment. The tracheoscopic cryobiopsy offers a novel, safe, and efficient tool for identifying pulmonary interstitial illness since it is less invasive, safe, and can provide more high-quality histological specimens. Cryointerventional treatment can be used on kids in tiny age groups because of the development of cryoprobes as small as 1.1 mm [28].

## 3. The Feasibility and Safety of Bronchoscopy with Cryotherapy

Due to its novelty, data on cryotherapy in the pediatric population is still scarce. A prospective multicenter study using bronchoscopic cryotherapy with indications for biopsy, airway patency restoration, and the aspiration of foreign bodies demonstrated an overall success rate of 93%. Transbronchial biopsies provided a diagnostic yield of 96%. Cryobiopsy is a good alternative to the widely used forceps biopsy [28]. Cryotherapy via flexible bronchoscopy has been shown to be a safe and feasible method for cap-shaped bronchial foreign body extraction, even in pediatrics [76]. It is suggested to use a flexible bronchoscope for organic foreign body removal if unsuccessful with a conventional bronchoscope [77]. It is important to identify foreign bodies early to minimize granulation tissue development, which complicates foreign body removal [23]. Flexible bronchoscopic cryoextraction has been used successfully in a critically ill and anticoagulated neonate [78].

## 4. Possible Complications of Bronchoscopy with Cryotherapy

Different body tissues are more or less tolerant of freezing than others. Generally speaking, tissues with less water, such as fat, bone, and fibrous connective tissue, are more tolerant to freezing than tissues with more amazing water content, such as granulation tissue, mucosa, and skin [13]. The tracheal and bronchial walls comprise the mucosa, submucosa, cartilage ring, and connective tissue. Their histological traits determine their high tolerance for cryotherapy, and complications like scar stenosis, osteomalacia, and perforation are rare. Even though extensive sample studies in children are uncommon [68,79], tracheoscopic freezing has been shown to be very safe in significant sample studies in adults [73,80]. The following freezing problems have been documented in the literature: bleeding, hypoxemia, perforation, subcutaneous emphysema, pneumothorax, and pneumomediastinum [32,79,80,81,82].

Schramm et al. [28] found superficial mucosal bleeding in 46.4% of 69 cryotherapy procedures, bleeding that required local application of vasoactive substances in 15.9% of procedures, pneumothorax in 2.9% of procedures, and bronchospasm in 1.4% of procedures. Chandra et al. found mild superficial bleeding in nine cases and pneumothorax in one case of cryobiopsy performed on the lung in 12 children [75]. According to the type, amount, and severity of the expression, the pneumothorax should be treated, which may include oxygen surveillance, chest closure drainage, or surgical intervention. The therapy of bleeding should be performed following the volume and severity of the bleeding and may involve the use of cold saline locally, local hemostatic pharmaceuticals, surgical intervention, pulmonary vascular interventional hemostasis, and surgical intervention.

## 5. Conclusions

Cryotherapy is a promising technique with a broad range of medical indications. Bronchoscopy cryotherapy application in the pediatric population is safe and possesses good efficacy. The application scope of flexible bronchoscopy cryotherapy in pediatrics is similar to that in adults, and it is mainly used in airway obstructive diseases. However, airway obstruction in children is primarily caused by benign conditions such as benign airway stenosis, softening, external pressure, or space, with very little involvement from malignant tumors. Interventional treatment in children seeks to provide long-lasting symptomatic alleviation, unlike interventional treatment in adults, which was primarily palliative treatment with airway cancer blockage. Therefore, interventional therapy, prevention, treatment, and the prognosis of long-term complications are issues that should be carefully considered in pediatric applications.

## Data Availability

The data presented in this study are available upon request from the corresponding author. The data are not publicly available due to restrictions regarding privacy or ethical concerns.

## Supplementary Materials

The following supporting information can be downloaded at: https://www.mdpi.com/article/10.3390/children11091130/s1, Video S1: Bronchoscopic cryotherapy procedure.
